# Distribution of HLA‐DQ risk genotypes for celiac disease in Ethiopian children

**DOI:** 10.1111/tan.14119

**Published:** 2020-10-30

**Authors:** Adugna N. Gudeta, Anita Ramelius, Taye T. Balcha, Alemayehu Girma, Jorma Ilonen, Daniel Agardh

**Affiliations:** ^1^ Department of Clinical Sciences Lund University Malmö Sweden; ^2^ Clinical Infection Medicine, Department of Translational Medicine Lund University Malmö Sweden; ^3^ Adama Hospital Medical College Adama Ethiopia; ^4^ Immunogenetics Laboratory, Institute of Biomedicine University of Turku Turku Finland

**Keywords:** celiac disease, children, Ethiopia, HLA‐DQ, Sweden

## Abstract

Most patients with celiac disease are positive for either HLA‐DQA1*05:01‐DQB1*02 (DQ2.5) or DQA1*03:01‐DQB1*03:02 (DQ8). Remaining few patients are usually DQA1*02:01‐DQB1*02 (DQ2.2) carriers. Screenings of populations with high frequencies of these HLA‐DQA1‐DQB1 haplotypes report a 1% to 3% celiac disease prevalence. The aim was to determine the prevalence of HLA‐DQ risk haplotypes for celiac disease in Ethiopian children. Dried blood spots collected from 1193 children from the Oromia regional state of Ethiopia were genotyped for HLA‐DQA1 and DQB1 genotyping using an asymmetric polymerase chain reaction (PCR) and a subsequent hybridization of allele‐specific probes. As references, 2000 previously HLA‐genotyped children randomly selected from the general population in Sweden were included. DQ2.2 was the most common haplotype and found in 15.3% of Ethiopian children, which was higher compared with 6.7% of Swedish references (*P* < .0001). Opposed to this finding, DQ2.5 and DQ8 occurred in 9.7% and 6.8% of Ethiopian children, which were less frequent compared with 12.8% and 13.1% of Swedish references, respectively (*P* < .0001). The DQ2.5‐*trans* genotype encoded by DQA1*05‐DQB1*03:01 in combination with DQ2.2 occurred in 3.6% of Ethiopian children, which was higher compared with 1.3% of Swedish references (*P* < .0001). However, when children with moderate high to very high‐risk HLA genotypes were grouped together, there was no difference between Ethiopian children and Swedish references (27.4% vs 29.0%) (*P* = .3504). The frequency of HLA risk haplotypes for celiac disease is very similar in Ethiopian and Swedish children. This finding of importance will be useful in future screening of children for celiac disease in Ethiopia.

## 1 INTRODUCTION

Knowing the distribution of HLA genotypes has proven to be useful for the prediction of celiac disease in the general population^1^ Celiac disease is an immune disorder strongly associated with the HLA‐DQA1*05:01‐DQB1*02:01 (DQ2.5) haplotype and to a lesser extent DQA1*03:01‐DQB1*03:02 (DQ8) haplotype. Individuals carrying at least one copy of the risk molecule DQ2.5 and/or DQ8 could account for more than 90% celiac disease.^2^, ^3^, ^4^ Most cases of celiac disease without either of these two haplotypes are positive for the DQA1*02:01‐DQB1*02:02 (DQ2.2) haplotype.^5^, ^6^ Subjects positive for the DQ2.2 and DQA1*05‐DQB1*03:01 haplotypes can also form DQ2.5 molecule in *trans* position, encoded by the DQA1*05 and DQB1*02 alleles in different parental chromosomes.^7^ Individuals being homozygote for DQ2.5 and those carrying the DQ2.5/DQ2.2 genotype are at a higher risk than those with only one DQ2.5 haplotype. Similarly, homozygosity for DQ2.2 increases the risk over one haplotype.^8^, ^9^ In addition to the genetic factors also various environmental factors are supposed to contribute to the disease risk.^10^

Celiac disease is particularly common in the Nordic countries affecting 2% to 3% of the general population^11^, ^12^, ^13^ and often co‐occurs in the same individual with type 1 diabetes.^14^, ^15^ A plausible explanation for the high prevalence there is the high proportion of HLA‐DQ2.5 and DQ8 carriers with susceptibility for the celiac disease and found in approximately 40% of the general population.^16^ Autoimmune diseases, like celiac disease, are expected to be rare in many developing countries. The variation in the distribution of HLA‐DQ2.5 and DQ8 among the general population may explain the difference in the prevalence between different geographical regions. In Western Europe, the Middle East and North Africa, where the prevalence of the celiac disease is high, the distribution of HLA‐DQ2.5 is reaching 20% and that of DQ8 in 10% in some regions compared with 10% respective 5% in Southeast Asia and China, where celiac disease is less frequent.^17^, ^18^ In contrast to most African countries, some parts of North Africa have a high prevalence of celiac disease (5.6%), which is partly explained by a very high frequency of DQ2.5 carriers. On the other hand, the prevalence of HLA‐DQ2.5 and DQ8 as well as celiac disease is still mainly unknown in sub‐Saharan African countries including Ethiopia.

Ethiopia is one of the developing sub‐Saharan countries with a population of approximately 100 million inhabitants with diversified ethnicities, which makes it the second‐most populated country in Africa after Nigeria. To our knowledge, only one previous small study has estimated the frequency of HLA genotypes in the Ethiopian general population and showed a proportion of HLA susceptible individuals in Ethiopia comparable with Northern Europe.^19^ The aim of the present study was to extend those previous investigations to determine the distribution of HLA‐DQ haplotypes and genotypes in an Ethiopian birth cohort and compare the prevalence with a HLA genotyped Swedish birth cohort prospectively followed for type 1 diabetes and celiac disease.

## 2 MATERIALS AND METHODS

### 2.1 Study population

The Traditional Ethiopian Food (TEF) study is an observational prospective screening study that follows a birth cohort for a celiac disease selected from the general population at three health centers in Adama located in the Oromia regional state, which is the largest of the nine regional states in Ethiopia. The overall goal of the TEF study is to determine the prevalence of celiac disease, and moreover, to identify what are the genetic and environmental factors that trigger or protect Ethiopian children from celiac disease. After informed consent by the guardian, a drop of blood from a finger stick was collected from the child on a filter paper at 9 months of age. Children enrolled in the study are followed annually with yearly visits and collection of blood samples between 24 months and 4 years of age. Between 1 April 2018 and 30 October 2019, parents of 1389 children were asked to participate of whom 1253 (90.0%) accepted participation and 1193 of 1253 (95.2%) were successfully HLA genotyped. Included for comparisons were 2000 previously HLA‐genotyped Swedish children randomly selected from 35 690 newborns in the Diabetes Prediction in Skåne (DiPiS) study, a longitudinal study with the main aim to predict type 1 diabetes and celiac disease in the general population.^20^, ^21^

### 2.2 HLA genotyping

HLA typing was performed at the Immunogenetics Laboratory at the University of Turku, Finland as described in the previous studies.^22^, ^23^ After a “full‐house” HLA‐DQB1 typing presence of DQA1*02:01, DQA1*03 and DQA1*05 were genotyped when informative for deducing common haplotypes. In the same manner, the genotypes HLA‐DQA1 and HLA‐DQB1 genotypes were determined for the DiPiS study by PCR and sequence‐specific probes as described in the previous study.^24^ In the DiPiS study series, only a part of DQB1*03:01 positive samples were genotyped for DQA1*03 and DQA1*05 (N = 77) and also only a part of DQB1*03:03 positive samples for DQBA1*02:01 and DQA1*03 (N = 67). Distribution of haplotypes in these samples were used for estimation their proportion in the whole cohort. HLA risk classifications for celiac disease are summarized in Table 1 and were stratified according to having very low risk to very high risk as described previously.^8^, ^21^, ^25^, ^26^, ^27^

**TABLE 1:** Classification of HLA risk genotypes for celiac disease associated according to different DQ molecule combinations

Celiac disease risk	HLA‐DQ genotype status
Very high risk	DQ2.5/DQ2.5
	DQ2.5/DQ2.2
Moderate risk	DQ2.5/DQ8
	DQ2.5_*trans*
	DQ2.5/X
	DQ2.2/DQ2.2
	DQ8/DQ8
	DQ8/DQ2.2
Low risk	DQ2.2/X
	DQ8/X
Very low risk	DQX/X

*Note*: Alleles encoding various molecules: DQA1*02‐DQB1*02, DQ2.2; DQA1*05‐DQB1*02, DQ2.5; DQA1*03‐DQB1*03:02, DQ8; other DQA1‐DQB1 combinations, X.

### 2.3 Ethical statement

This study was approved by the institutional review board of Armauer Hansen Research Institute (P028/17), National Research Ethics Committee of Ethiopia (Ref. No. 3.10/16/2018), and the ethical committee Lund University (Pro.No.2017/3). A supportive letter was obtained from Oromia national regional state health offices of Ethiopia. Consent was obtained from mothers or legal guardians of each study children. All samples and related questioners were processed and kept by codes without any personnel identity. The DiPiS study was approved by the ethical committee Lund University (LU 490‐99).

### 2.4 Statistical analysis

A descriptive statistic by using SPSS for Windows, version 25; SPSS Inc. Chicago, Illinois was used to calculate the frequencies of the HLA haplotypes, and differences between groups were estimated by the chi‐squared test. Bonferroni's test was used for multiple comparisons. A *P*‐value <.05 was considered statistically significant.

## 3 RESULTS

### 3.1 HLA DQA1‐DQB1 haplotypes in Ethiopian children vs Swedish references

The DQ2.2 haplotype was the most common among Ethiopian children and found in 15.3% (364/2386) of haplotypes compared with 6.7% (269/4000) of Swedish references (*P* < .0001; Table 2).

**TABLE 2:** Distribution of HLA DQA1‐DQB1 haplotypes among 1193 Ethiopian children and in 2000 Swedish children used as references

HLA haplotype	Ethiopian cohort		Swedish references		*P*‐value
	N	%	N	%	
DQA1*02:01‐DQB1*02 (DQ2.2)	364	15.3	269	6.7	<0.0001
(DQA1*01)‐DQB1*05:01	341	14.3	434	10.9	<0.0001
DQA1*05‐DQB1*03:01 (DQ7.5)	285	12.0	366	9.1	0.0004
(DQA1*01)‐DQB1*06:02	239	10.0	560	14.0	<0.0001
(DQA1*01)‐DQB1*06:04	238	10.0	244	6.1	<0.0001
DQA1*05‐DQB1*02 (DQ2.5)	232	9.7	513	12.8	0.0002
DQA1*03‐DQB1*03:02 (DQ8)	161	6.8	523	13.1	<0.0001
(DQA1*01)‐DQB1*06:03	115	4.8	234	5.9	0.0797
DQA1*03‐DQB1*02 (DQ2.3)	112	4.7	4	0.1	<0.0001
(DQA1*01)‐DQB1*06:09	95	4.0	23	0.6	<0.0001
(DQA1*03/*04)‐DQB1*04	80	6.7	145	3.6	0.5683
DQA1*02:01‐DQB1*03:03	52	2.2	88	2.2	0.9566
DQA1*03‐DQB1*03:01	30	1.3	246	6.2	<0.0001
(DQA1*01)‐DQB1*05:03	17	0.7	81	2.0	<0.0001
DQA1*03‐DQB1*03:03	8	0.3	67	1.7	<0.0001
(DQA1*01)‐DQB1*05:02/04	5	0.2	65	1.6	<0.0001
Other haplotypes	12	0.5	138	3.5	<0.00001

The two haplotypes associated most strongly with CD susceptibility, DQ2.5 (DQA1*05‐DQB1*02) and DQ8 (DQA1*03‐DQB1*03:02), were found in 9.7% (232/2386) and 6.8% (161/2386) of Ethiopian haplotypes compared with 12.8% (513/4000) and 13.1% (523/4000) of Swedish references, respectively (*P* < .001). The DQ7.5 (DQA1*05‐DQB1*03:01) haplotype was found in 12.0% (285/2386) of Ethiopian haplotypes compared with 9.1% (366/4000) of Swedish references, respectively (*P* < .001). The DQ2.3 (DQA1*03‐DQB1*02) haplotype was common in Ethiopia and found in 4.7% (112/2386) of haplotypes but found in few of the Swedish references (0.1% [4/4000]; *P* < .0001).

### 3.2 CD‐associated HLA genotypes in Ethiopian children vs Swedish references

Among Ethiopian children, 12.1% (144/1193) were positive for DQ2.5/X and 7.4% (89/1193) for DQ8/X, which were both lower compared with 17.1% (342/2000) and 17.5% (349/2000) of Swedish references, respectively (*P* < .0001). Similarly, were DQ2.5/DQ8 heterozygotes less common in Ethiopian children compared with Swedish references (Table 3). However, due to the high frequency of the DQ2.2 and DQ7.5 haplotypes was the genotype encoding DQ2.5_*trans* molecule more common among Ethiopian children as well as DQ2.2/X and DQ2.2/DQ2.2 combinations. There was no difference in frequency of the two HLA genotypes associated with very high risk, DQ2.2/DQ2.5 and DQ2.5/DQ2.5, likewise also DQ2.2/DQ8 and DQ8 homozygotes when Ethiopian children were compared with Swedish references (Table 3).

**TABLE 3:** Distribution of HLA genotypes among 1193 Ethiopian children and in with 2000 Swedish children used as references

	Ethiopian cohort		Swedish references		*P*‐value
HLA‐DQ genotype	N	%	N	%	
DQ2.2/X	200	16.8	189	9.5	<0.0001
DQ2.5/X	144	12.1	342	17.1	0.00025
DQ8/X	89	7.4	349	17.5	<0.0001
DQ2.5_*trans*	43	3.6	26	1.3	<0.0001
DQ2.2/DQ2.2	39	3.3	4	0.2	<0.0001
DQ2.5/DQ2.5	26	2.2	34	1.7	0.33681
DQ2.5/DQ2.2	22	1.8	33	1.7	0.68474
DQ2.2/DQ8	21	1.8	38	1.9	0.77771
DQ8/DQ8	18	1.5	32	1.6	0.84147
DQ2.5/DQ8	14	1.2	70	3.5	<0.0001
X/X	577	48.4	883	44.2	0.0523

*Note*: Alleles encoding various molecules: DQA1*02:01‐DQB1*02, DQ2.2; DQA1*05‐DQB1*02, DQ2.5; DQA1*05‐DQB1*03:01 together with DQA1*02:01‐DQB1*02, DQ2.5_*trans*; DQA1*03‐DQB1*03:02, DQ8; Other *DQA1‐DQB1* haplotypes including DQA1*05‐DQB1*03:01 in other genotype combinations, X.

**FIGURE 1:**
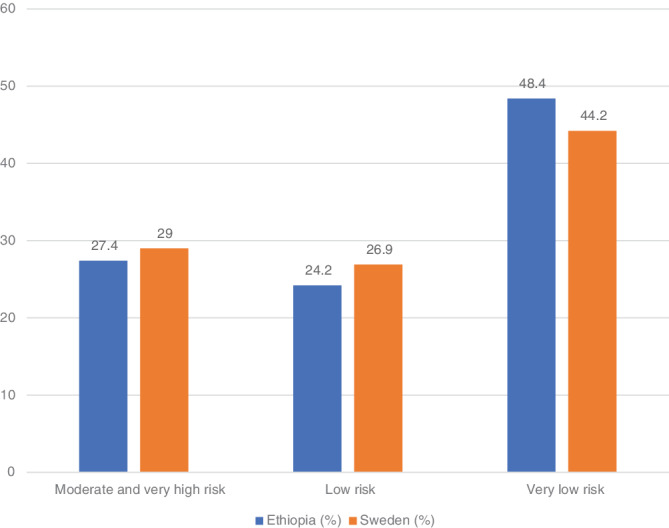
Comparison of HLA‐DQ genotypes by celiac disease risk among Ethiopian children vs Swedish children used as the reference group

When children with moderate high to very high‐risk HLA genotypes were grouped together (Table 1), there was no difference between Ethiopian children and the Swedish references (27.4% [327/1193] vs 29.0% [579/2000]; *P* = .3504; Figure 1). The overall frequency of having very low risk was 48.4% (577/1193) in Ethiopian children, which was slightly higher compared with 44.2% (884/2000) of Swedish references (*P* = .0222; Figure 1).

## 4 DISCUSSION

This study estimated the prevalence of HLA‐DQ risk haplotypes and genotypes for celiac disease in Ethiopian children and compared the frequencies of a previously HLA‐genotyped Swedish birth cohort. The frequency of DQ2.5 haplotype, which is found in most celiac disease patients worldwide^9^, was higher in Swedish references than in Ethiopian as well as the DQ8 haplotype, found in most patients without DQ2.5. In contrast, the most frequent haplotype in Ethiopian children was the DQ2.2 haplotype, which was higher compared with Swedish references. Only few patients with CD are DQ2.2 positive without having any of DQ2.5 or DQ8, but in combination of DQ7.5 and DQ2.2 the DQ2.5 molecule can still be encoded in *trans* position. In addition to DQ2.2, Ethiopian children had high frequency of the DQA1*05‐DQB1*03:01 haplotype compared with Swedish references. This overall prevalence of children with DQ2.5 in *trans* position was thus nearly three times as high in Ethiopian compared with Swedish references. When comparing risk molecule frequencies with the Swedish references, the frequencies of the DQ2.2 and DQ2.5 *trans* molecule were therefore in fact higher among the Ethiopian children, although the opposite was true for DQ2.5 *cis* and DQ8. In line with our results, another study also found a high frequency of DQ2.2 among the Ethiopian Oromo population (15.5%).^19^

The biggest difference in haplotype distribution between the Ethiopian and Swedish populations was conferred by DQ2.3, which is very rare in Northern Europe but found with relatively high frequency in many Mediterranean countries and in Africa in linkage with several DR alleles and associated with increased risk of type 1 diabetes.^28^, ^29^, ^30^ Although the DQ2.3 association with type 1 diabetes has been well studied, it is still unknown whether it contributes to an increased risk of CD due to lack of studies. In type 1 diabetes, it is also known to be encoded in *trans* position in patients carrying the high‐risk‐associated DQ2/DQ8 genotype. Interestingly, *trans* encoded DQ2.3 molecules were able to present a deamidated gluten peptide epitopes even more efficiently than the DQ2.5 and DQ8 molecules indicating a possible role in the pathogenesis of the celiac disease.^31^

Differences in frequency of HLA‐DQ risk haplotypes among populations may partly explain differences in reported prevalence of celiac disease and/or type 1 diabetes.^32^ In the present study, the overall frequency of HLA risk was quite similar between Ethiopian and Swedish children when including all variants of DQ2 (including DQ2.5 in *cis* or *trans* position and DQ2.2). The frequencies in our Ethiopian cohort were comparable with results from studies in Algeria (28.3%), Morocco (25%), Libya (34%) and northern India (31.9%).^32^, ^33^, ^34^ On the other hand, the frequency of DQ2 was higher than the results of studies from Iran (20%), Turkey (18%), Southern India (12.78%), Rwanda (15.5%), Tanzania (13.5%) and Cameroon (7.0%),^32^, ^33^, ^34^ but lower compared with a study on the Saharawi population (39%), where the prevalence of the celiac disease has been estimated to be the highest in the world.^35^

Our study also showed that the DQ8 haplotype was less frequently found among the Ethiopian children as compared with the Swedish references (6.8% vs 13.1%), but comparable with study findings from Japan (7.6%), Sardinia (5%)^33^ and 8.9% of Caucasians in the United States.^36^ In addition, we found a higher distribution of DQ8 than in South Africa (2.8%), Saharawi (2.7%), Algeria (2.2%), Italy (2%) and Cameroon (0.6%).^34^ Compared with our study, higher frequencies in the review by Alarida were reported from Mexico (28.3%), Turkey (22%), North India (15.6%) and Iran (12%).^34^

Our findings indicate that more than half (51.6%) of the Ethiopian population is carrying any of the DQ2.5, DQ2.2 and DQ8 haplotypes, which was comparable with the Swedish references (55.9%) and other previous studies performed on the general population in Australia (55.9%),^37^ Iran (58%),^18^ Saudi Arabia (52.7%),^25^ but slightly higher than Brazil (43.7%)^38^, ^39^ and Denmark (47.7%).^40^ Although this study is the largest HLA‐genotyped Ethiopian population to date, it is limited by the fact that children included in the present study were enrolled from only one region and therefore may not represent the whole population in Ethiopia. We can therefore only speculate that our results might partly explain the low reported prevalence of the celiac disease in Ethiopia as compared with Sweden,^11^ albeit the true prevalence in the general population is still unknown.

In conclusion, the present study showed that the overall distribution of HLA risk haplotypes for celiac disease is very similar in Ethiopian compared with Swedish children. This new knowledge might be useful in future screening of children from the general population for celiac disease in Ethiopia.

## CONFLICT OF INTEREST

The authors have declared no conflicting interests.

## AUTHOR CONTRIBUTIONS

A.N.G.: substantial contribution to the conception or design of the project, data curation, analysis or interpretation, project administration, drafting and revising the manuscript critically. A.R.: editing and revising the manuscript. T.T.B.: contribution to the conception of the project and revising the manuscript. A.G.: conceptualization and review and editing. J.I.: Substantial contribution to the conception or design of the project, performed HLA typing, analysis, revising the manuscript critically and approve the version to be published. D.A.: substantial contribution to the conception or design of the project, interpretation of data for the work, project administration, resources and supervision, revising the manuscript critically and approve the version to be published.

## Data Availability

The data that support the findings of this study are available from the corresponding author upon reasonable request.
